# How to Change the Role of Allogeneic Hematopoietic Cell Transplantation in Adults with B-Cell Acute Lymphoblastic Leukemia

**DOI:** 10.3390/cimb48040351

**Published:** 2026-03-27

**Authors:** Martina Canichella, Paolo de Fabritiis

**Affiliations:** 1 Hematology, St. Eugenio Hospital, ASL Roma2, 00144 Rome, Italy; 2 Department of Biomedicina e Prevenzione, Tor Vergata University, 00133 Rome, Italy

**Keywords:** allogeneic hematopoietic stem cell transplantation, B-cell acute lymphoblastic leukemia, blinatumomab, chimeric-antigen receptor T-cell, inotuzumab ozogamicin, measurable residual disease, tyrosine kinase inhibitors

## Abstract

Allogeneic hematopoietic stem cell transplantation (allo-HSCT) has long constituted a cornerstone of post-remission consolidation therapy for adults with high-risk B-cell acute lymphoblastic leukemia (B-ALL), offering potent graft-versus-leukemia activity at the expense of significant treatment-related toxicity (TRT) and non-relapse mortality (NRM). Over the past two decades, however, outcomes following allo-HSCT have improved substantially. This progress has been driven primarily by a marked reduction in NRM, translating into improved overall survival (OS), as consistently documented by large cooperative group analyses and single-center series. Advances in supportive care, infectious prophylaxis, donor selection, and graft-versus-host disease (GvHD) prevention have contributed substantially to this improvement. In parallel, transplant decision-making has been profoundly reshaped by refined disease biology-based risk stratification and the systematic evaluation of measurable residual disease (MRD). Moreover, the advent of highly effective immunotherapeutic approaches—including blinatumomab, inotuzumab ozogamicin, and chimeric antigen receptor (CAR) T-cell therapies—has enabled the achievement of deeper molecular remissions prior to transplantation, both in first and subsequent complete remissions. Taken together, these developments have shifted allo-HSCT from a widely applied strategy to a more individualized, risk-adapted therapeutic approach. This review examines how the indications, timing, and objectives of allo-HSCT are evolving in the contemporary treatment landscape of adult B-ALL, with particular emphasis on Philadelphia chromosome–negative, Philadelphia-like, and Philadelphia chromosome–positive disease subsets.

## 1. Introduction

**Figure 1:**
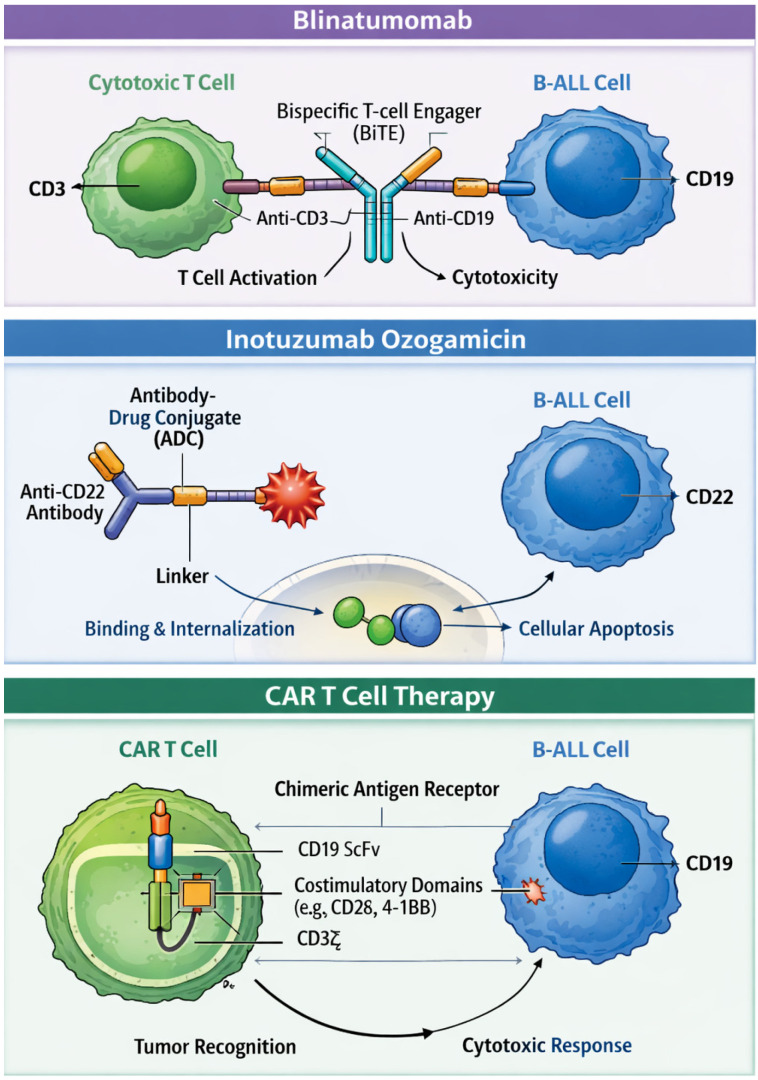
Distinct immunotherapeutic mechanisms of action targeting B-cell acute lymphoblastic leukemia: blinatumomab, inotuzumab ozogamicin, and CD19-directed CAR-T cells.

Allogeneic stem cell transplantation (allo-HSCT) has traditionally played a central role as a consolidation strategy for adult patients with high-risk B-cell acute lymphoblastic leukemia (B-ALL). Allo-HSCT offered the most effective anti-leukemic control in this setting, albeit at the cost of substantial treatment-related toxicity (TRT) and non-relapse mortality (NRM). However, in the last two decades, a progressive and clinically meaningful improvement in outcome has been observed among patients with B-ALL undergoing allo-HSCT. This evolution has been driven primarily by a marked reduction in NRM, which has translated into a significant improvement in overall survival (OS). Large study groups clearly document this trend. Giebel and colleagues, on behalf of the European Bone Marrow Transplantation (EBMT) group, showed a progressive reduction in NRM over time, declining to approximately 15–20%, accompanied by parallel improvements in leukemia-free survival (LFS) and OS [1]. Notably, infectious complications—particularly invasive fungal diseases—have historically represented a major cause of morbidity and mortality after allo-HSCT, especially among patients with GvHD; although the introduction of modern antifungal prophylaxis has significantly reduced this burden, these complications remain clinically relevant and continue to contribute to transplant-related mortality [2]. The reduction in NRM is largely attributable to advances in supportive care, improved infectious prophylaxis, both viral and fungal; more accurate donor selection strategies; and more effective prevention and management of graft-versus-host disease (GvHD) [2]. Similarly, the MD Anderson Cancer Center (MDACC) demonstrated substantial survival over the past four decades, with a median 5-year OS increase from 24% (median survival 15 months) in patients diagnosed between 1983 and 1989 to 66% (median survival 131 months) in those treated between 2010 and 2022 [3]. Beyond these improvements in transplant procedures, additional factors have recently contributed to reshaping transplant decisions [4]. These advances include a more precise patient selection through refined risk stratification, based on deeper characterization of biological disease signatures and systematic assessment of measurable minimal residual disease (MRD). In parallel, the introduction of highly effective immunotherapies—such as the bispecific antibody blinatumomab, the antibody–drug conjugate inotuzumab ozogamicin (InO), and cellular therapy with chimeric antigen receptor (CAR) T-cell therapies—has enabled deeper molecular remission before transplantation in both first and second complete remission (CR1 and CR2). Taken together, these developments are profoundly changing the role of allo-HSCT in adult B-ALL (Figure 1) [5]. This review aims to illustrate how the indication, timing, and objectives of allo-HSCT are evolving in the contemporary treatment landscape. First, we provide a brief overview of the major advances that have led to improvements in allo-HSCT techniques and transplant platforms. Then, we discuss how the role of allo-HSCT is changing across the three main B-ALL subtypes: Philadelphia (Ph)-negative ALL, Ph-like ALL, and Ph-positive ALL.

## 2. Improvements in Allo-HSCT Procedure

Substantial advances in the technical execution of allo-HSCT have been achieved over the past two decades [5]. Progressive refinements in several aspects have translated into a meaningful reduction in TRT and NRM, while preserving the graft-versus-leukemia (GvL) effect that represents the curative potential of transplantation [6,7]. These developments primarily involve optimization of conditioning regimens, refinement of GvHD prophylaxis and management, improved donor selection with expanded donor availability, and major advances in supportive care. Transplant strategies are now increasingly individualized, integrating patient-related factors such as age, functional status, and comorbidities with disease-specific variables, including biological class of risk and MRD status. Importantly, the MRD status pre-allo-HSCT dramatically influenced the outcome. In adult patients with Ph-negative ALL treated with the pediatric-inspired Gruppo Italiano Malattie EMatologiche dell’adulto (GIMEMA) LAL1913 protocol who underwent allo-HSCT, 3-year OS and disease-free survival (DFS) were 68% and 60%, respectively. MRD-negative status at transplant was associated with superior outcome (3-year OS 80% vs. 47%; DFS 70% vs. 41%), and even in second remission, MRD negativity conferred better survival (OS 60%; DFS 56% vs. 13% in MRD-positive). Indeed, NRM at 3 years remained below 20% [8]. Within this framework, ongoing efforts aim to better define patient subsets who may benefit from reduced-intensity conditioning (RIC), while clarifying scenarios in which myeloablative conditioning (MAC) remains necessary to achieve durable disease control. At the same time, advances in conditioning regimens have significantly contributed to improving allo-HSCT outcomes in both myeloid malignancies and B-ALL, primarily through the reduction of TRM. In this regard, the introduction of novel agents such as treosulfan has expanded the therapeutic landscape, offering effective myeloablation with a more favorable toxicity profile and thereby further optimizing the balance between disease control and TRM. The improved understanding of the pathogenic mechanisms underlying transplant-related complications such as SOS/VOD has clarified the role of genetic susceptibility—including in patients treated with InO—and supported risk-adapted prophylactic strategies, such as the use of defibrotide [9,10]. In parallel, the implementation of high-resolution HLA typing; the widespread use of alternative donors—including matched unrelated and haploidentical donors; and the adoption of post-transplant cyclophosphamide-based GvHD prophylaxis have substantially opened up access to allo-HSCT. In B-ALL, total-body irradiation (TBI) remains a cornerstone of conditioning due to its potent antileukemic activity and its role in promoting engraftment [11]. Meta-analyses consistently demonstrate superior overall and progression-free survival (PFS), with lower relapse rates, for TBI-based myeloablative regimens compared with chemotherapy-only approaches. More recently, advances in radiation delivery, such as intensity-modulated total-body or total-marrow irradiation, have been developed to maintain antileukemic efficacy while mitigating acute and long-term toxicities [12]. Importantly, improvements in post-transplant outcomes have been increasingly attributed to better timing of the procedure, with best survival and relapse data seen when transplant is performed in the context of pre-transplant MRD negativity, underscoring the prognostic and therapeutic value of achieving deep remission before HCT and translating into enhanced long-term quality of life [13].

## 3. B-ALL Subtype

The diagnosis of B-ALL is primarily based on morphological evaluation, supported and refined by immunophenotypic characterization. According to the World Health Organization (WHO) 5th edition classification, eleven distinct molecular and cytogenetic subtypes are currently recognized, including the subgroup of Ph-like B-ALL which will be discussed separately below [14]. Each of these subtypes is associated with a specific prognostic impact, as summarized in Table 1.

Indeed, the International Consensus Classification (ICC) of ALL, which is primarily a genomics-based classification, has highlighted—through the application of increasingly advanced molecular techniques—the existence of additional B-ALL subtypes associated with recurrent gene fusions or somatic mutations [15]. However, it should be noted that within Ph-negative B-ALL, a proportion of cases still lack an identifiable cytogenetic or molecular signature (roughly 50% of B-ALL) and currently represent the true standard-risk group. Importantly, prognostic stratification of B-ALL does not rely exclusively on cytogenetic and molecular features assessed at diagnosis but also incorporates patient-related factors and treatment response parameters, including early response and measurable residual disease (MRD) assessment, as discussed below.

### Prognostic Stratification and the Role of MRD

Over the past two decades, risk stratification in B-ALL has undergone continuous refinement, aimed at improving prognostic implication and driving therapeutic decisions. The stratification models integrate three principal factors: patient-related (such as age, performance status (PS), and white blood cell (WBC) count at diagnosis), disease-related biological features (including cytogenetic and molecular abnormalities), and response to treatment, with particular attention on MRD status [16]. MRD can be evaluated using different methodological approaches, including multiparameter flow cytometry (MFC), quantitative polymerase chain reaction (qPCR), and next-generation sequencing (NGS), each characterized by distinct analytical sensitivity and applicability (Table 2). MRD is most commonly assessed by MFC and qPCR, each with distinct strengths and limitations. MFC is widely applicable (>90%), rapid, and standardized, but has lower sensitivity and is affected by sample quality, phenotypic shifts, and operator dependence. Molecular approaches (fusion genes, IG/TR PCR, and NGS) offer higher sensitivity and standardization in selected settings, but are limited by applicability, clonal evolution, nucleic acid quantity, and—especially for NGS—lack of harmonization and routine clinical adoption and high cost [17]. Clinical variables such as older age and elevated WBC counts have long been recognized as adverse prognostic factors, whereas specific genetic aberrations further delineate high-risk subsets (Table 1). Importantly, MRD assessment has emerged as the most powerful prognostic marker across age groups and treatment modalities and is now widely recommended in all patients to optimize risk classification and therapeutic programs [17,18]. A systematic comparison of risk stratification models across study groups highlights the central role of MRD: in a European survey, MRD was the only factor consistently used by national groups to define high-risk ALL and to make decisions regarding transplant allocation, whereas adverse genetics, although important, did not achieve the same level of consensus [19]. The prognostic value of MRD is further illustrated by its integration with cytogenetic and molecular profiles in risk algorithms, where early MRD clearance correlates with favorable outcome and can support de-intensification of therapy [20]. In contrast, patients with persistent or recurrent MRD typically warrant treatment intensification or alternative strategies such as allo-HSCT or targeted agents. Importantly, MRD status also modifies transplant decisions [21]. Standard-risk patients by diagnostic criteria may be reclassified as high-risk based on MRD persistence at key early treatment time points, thereby becoming candidates for allo-HSCT [17,18]. This dynamic risk reassessment underscores the pivotal role of MRD in shaping therapeutic pathways and optimizing outcomes in contemporary B-ALL management. Overall, refined prognostic stratification has become a cornerstone of modern B-ALL management, allowing treatment intensity to be tailored according to relapse risk. Among all prognostic tools, MRD assessment represents the most powerful and dynamic factor, as patients classified as standard risk at diagnosis may be re-allocated to a high-risk category based on persistent MRD positivity at predefined time points, thereby acquiring eligibility for allogeneic stem cell transplantation.

**Table 1:** Prognostic factors in adult B-ALL (adapted from Gökbuget et al., 2024 [17]).

Prognostic Factors	Factors	Clinical Relevance
Patient-related factors	Age at diagnosis	Increasing age is associated with inferior outcomes and higher treatment-related toxicity
	Performance status/comorbidities	Influence treatment intensity and transplant eligibility
	White blood cell count at diagnosis	High WBC count correlates with increased relapse risk
	Central nervous system involvement	Associated with poorer prognosis and need for treatment intensification
Disease-related factors	Cytogenetic abnormalities	High-risk lesions (e.g., KMT2A rearrangements, hypodiploidy, and complex karyotype) predict inferior survival
	Molecular genetic features	Ph-like profile, IKZF1 alterations, and other adverse mutations refine risk assessment
	Immunophenotypic features	Lineage and aberrant antigen expression may influence prognosis and treatment response
Response-related factors	Early treatment response	Speed of blast clearance reflects chemosensitivity
	MRD	Most powerful prognostic factor across all risk groups
	MRD status at defined time points	Persistent MRD positivity identifies patients at high risk of relapse
	MRD kinetics	Failure to achieve or maintain MRD negativity predicts poor outcome

MRD, measurable residual disease; WBC, white blood cell; Ph, Philadelphia.

**Table 2:** Different MRD assays in B-ALL.

Method	Target	Sensitivity	Advantages	Limitations
Multiparameter flow cytometry (MFC)	Leukemia-associated immunophenotypes (LAIP)	10^−4^	Rapid, widely available, applicable to most patients	Operator-dependent, immunophenotypic shifts
Real-time quantitative PCR (RQ-PCR)	IG/TR gene rearrangements	10^−5^	High sensitivity, standardized (EuroMRD)	Requires diagnostic material, not applicable to all cases
RQ-PCR	Fusion transcripts (e.g., BCR::ABL1)	10^−5^–10^−6^	Highly sensitive and specific	Applicable only to selected subtypes
Next-generation sequencing (NGS)	IG/TR rearrangements	10^−6^	Highest sensitivity, clonal evolution tracking	Cost, longer turnaround, limited standardization
Digital droplet PCR (ddPCR)	Gene rearrangements or mutations	10^−5^–10^−6^	Absolute quantification, high precision	Limited availability, assay-specific

## 4. Ph-Negative B-ALL

Multiple study groups demonstrated that patients with high-risk Ph-negative B-ALL —defined by adverse cytogenetic–molecular features or by persistent MRD positivity at the end of induction—experience an unfavorable prognosis. This high-risk subgroup is characterized by a significantly increased risk of relapse and mortality, resulting in limited DFS with conventional chemotherapy alone. For these patients, allo-HSCT performed in CR1 has been widely shown to represent the therapeutic strategy associated with improvements of outcome [22]. In patients with relapsed or refractory (R/R) Ph-negative B-cell ALL who achieve a CR2, two major factors have substantially contributed to the improvement of post-remission outcomes and, consequently, the consolidation of the role of allo-HSCT. First, the integration of MRD assessment into treatment algorithms has enabled more precise risk stratification and transplant timing, identifying patients who derive the greatest benefit from transplantation [23]. Second, the introduction of highly effective immunotherapeutic agents has significantly increased the proportion of patients achieving deep molecular remissions prior to allo-HSCT, thereby reducing relapse risk and improving post-transplant survival [24]. Across multiple studies, the MRD negativity has emerged as one of the strongest predictors of favorable outcome, being associated with significantly prolonged relapse-free survival and OS, irrespective of the specific methodology used for MRD detection.

## 5. Novel Therapies as a Bridge to Allo-HSCT

The introduction of novel immunotherapeutic approaches has contributed to reshaping the role of allo-HSCT in B-ALL, both by enabling deeper responses at relapse and by consolidating MRD negativity in the frontline setting. Below, we summarize the main results of clinical studies evaluating the use of these novel immunotherapeutic agents.

### 5.1. Blinatumomab

Blinatumomab is a bispecific T-cell engager (BiTE) that simultaneously binds CD3 on cytotoxic T lymphocytes and CD19 on B-lineage cells, leading to formation of an immunological synapse and T-cell activation independent of costimulatory signals. This interaction induces targeted cytotoxicity through perforin- and granzyme-mediated apoptosis, enabling serial killing of CD19-positive leukemic cells [25].

In adults with R/R B-ALL, blinatumomab is administered as a continuous intravenous infusion at 9 µg/day during the first 7 days of cycle 1, followed by 28 µg/day thereafter, with each cycle consisting of 28 days of infusion and a 14-day treatment-free interval. It was initially granted accelerated FDA approval in 2014 for R/R B-ALL, and subsequent regulatory extensions include treatment of MRD-positive B-ALL in CR1 or CR2 with MRD ≥ 0.1% [26]. More recently, blinatumomab was approved in the consolidation phase of frontline therapy for CD19-positive, Ph-negative B-ALL, driven by improved OS versus conventional chemotherapy [27]. The role of blinatumomab in inducing deep remission, thereby enabling patients to proceed to allo-HSCT with a lower disease burden, was already evident in early clinical trials and has subsequently been confirmed by real-world studies. This evidence consistently demonstrates that blinatumomab can achieve a high rate of MRD negativity, both in CR1 and CR2 facilitating bridging to transplant and contributing to improved post-transplant outcomes across the adult and pediatric population [28]. The pivotal Phase II BLAST trial investigated blinatumomab in adults with MRD-positive B-ALL in CR1 or CR2 and demonstrated that 78% of evaluable patients (88/113) achieved complete MRD negativity after one cycle of therapy, with 67% (74/110) of patients proceeding to allo-HSCT. Long-term follow-up showed a median OS of 36.5 months and median relapse-free survival (RFS) of 18.9 months, with MRD responders exhibiting significantly prolonged RFS (median 23.6 months vs. 5.7 months) and OS compared with non-responders. After blinatumomab and subsequently allo-HSCT, median survival was not reached, further underscoring the prognostic importance of achieving MRD negativity prior to transplant [29]. In the Phase III TOWER trial, adults with Ph-negative R/R B-ALL were randomized to blinatumomab versus standard salvage chemotherapy; blinatumomab significantly improved clinical outcomes, with a CR rate of ~44% versus ~25% with chemotherapy, and a median OS of 7.7 months compared with 4 months, supporting its use as an effective salvage therapy that can bridge to allo-HSCT. These findings have been confirmed also in a sub-analysis of TOWER which reported that, of the patients who ultimately underwent allo-HSCT during the study period, 65 patients in the blinatumomab arm proceeded to transplant, compared with 32 in the chemotherapy arm. Beyond these pivotal trials, retrospective and real-world cohorts have specifically evaluated blinatumomab, confirming these results [30]. In a single-center case series by Fu et al., incorporation of blinatumomab into a short-course conditioning regimen for three adult patients with MRD-positive B-ALL resulted in 100% MRD negativity pre-HSCT, and two of three patients (≈66.7%) remained disease-free at 2 years post-transplant, with no severe adverse events [31]. A large retrospective cohort from Princess Margaret Hospital found that pre-transplant blinatumomab in adult B-ALL was associated with significantly higher 2-year OS (65.4% vs. 45.6%) and graft-versus-host disease-free (GRFS) RFS (GRFS; 42.2% vs. 17.3%), along with markedly lower non-relapse mortality (3.2% vs. 43.0%), compared with patients not receiving blinatumomab [32]. In pediatric and young adult populations, blinatumomab also consistently enables successful bridging to transplant. In a cohort of 13 pediatric patients with R/R B-ALL, blinatumomab achieved an 85% overall response rate (ORR), with 10 of 13 patients (≈77%) attaining MRD negativity after one cycle and 11 of 13 (≈85%) proceeding to allo-HSCT. More extensive pediatric data from Llaurador et al. showed 100% MRD negativity (31/31) among treated children and young adults receiving blinatumomab prior to allo-HSCT, with significant improvements in leukemia-free survival versus chemotherapy controls [33]. Similarly, Algeri et al. reported that in 78 children and young adults treated with blinatumomab as last consolidation before allo-HSCT, ≈92% achieved MRD negativity pre-transplant, with 2-year DFS of 72.2%, OS of 89.2%, and a low NRM (2.6%) [34]. Collectively, these prospective and retrospective datasets demonstrate that blinatumomab reliably induces deep molecular remissions, increases rates of MRD negativity prior to conditioning, and is associated with favorable rates of transplant realization and post-allo-HSCT survival, firmly establishing its role as a well-tolerated and efficacious bridge to allo-HSCT in high-risk B-ALL (Table 3).

### 5.2. Inotuzumab Ozogamicin (InO)

InO is a CD22-directed antibody–drug conjugate composed of a humanized monoclonal antibody linked to the cytotoxic agent calicheamicin. Upon binding to CD22-positive B-lineage cells, the complex is internalized, releasing calicheamicin intracellularly, where it induces double-strand DNA breaks, cell cycle arrest, and apoptotic cell death. This mechanism results in potent, antigen-specific cytotoxicity independent of immune effector cell recruitment. In adults with relapsed or refractory B-cell acute lymphoblastic leukemia, InO is administered intravenously in 21- or 28-day cycles, with a total dose of 1.8 mg/m^2^ in cycle 1 (0.8 mg/m^2^ on day 1 and 0.5 mg/m^2^ on days 8 and 15), followed by 1.5 mg/m^2^ per cycle in subsequent cycles (0.5 mg/m^2^ on days 1, 8, and 15) [35,36]. It has been established as an effective immunotherapeutic agent in R/R B-ALL, demonstrating high rates CR and MRD negativity that facilitate subsequent allo-HSCT. The INO-VATE trial showed significantly higher CR/CRi and MRD-negative remission rates compared with standard chemotherapy, with post-transplant sinusoidal obstruction syndrome/veno-occlusive disease (SOS/VOD) cited as a significant toxicity associated with InO exposure, particularly when more than two cycles were administered or when conditioning regimens included dual alkylators [37]. Real-world registry analyses have corroborated these findings, observing that 14–18% of patients receiving InO prior to allo-HSCT developed SOS/VOD within 100 days post-transplant, consistent with clinical trial rates, underscoring the need for risk minimization strategies and careful timing of transplant [38].

More recent real-world cohort data further characterize InO as an effective bridge to allo-HSCT: in a retrospective analysis of 58 adult patients with R/R B-ALL treated with InO and proceeding to allo-HSCT, 84% achieved CR prior to transplant and 59% of MRD-evaluable patients achieved MRD negativity; median OS post-InO was 11.2 months, with 1- and 2-year OS rates of 50% and 36.7%, respectively; however, 29% experienced SOS/VOD after allo-HSCT with a high case fatality proportion among affected individuals [39]. Other retrospective series describe that InO (alone or combined with low-intensity chemotherapy) results in MRD clearance in a majority of responders and acts as a bridge to allo-HSCT—for example, in a real-world cohort treated with InO ± blinatumomab, the CR rate before first HSCT was 77.3%, with significantly improved 2-year OS (63.0% vs. 31.2%) and DFS (49.6% vs. 22.9%) compared with conventional salvage regimens, and with a trend toward lower NRM [40]. Given these risks, early clinical practice often advocated a longer interval of approximately 3 months between the last dose of InO and allo-HSCT to mitigate SOS/VOD. More recent expert consensus and registry analyses suggest that a shorter interval of 3–4 weeks may be feasible in selected patients if the number of InO cycles is limited (typically ≤2), dual-alkylator conditioning is avoided, liver function is closely monitored, and prophylactic measures such as ursodeoxycholic acid are implemented [40,41]. These data collectively demonstrate that InO, as well as blinatumomab, is an effective bridge to allo-HSCT, capable of inducing deep remissions and MRD negativity, but its clinical application requires deliberate timing and vigilant monitoring to minimize transplant-related hepatotoxicity (Table 4) [42].

### 5.3. CAR T-Cell Therapy

Chimeric antigen receptor T cells (CAR-T cells) are autologous T lymphocytes genetically engineered to express a synthetic receptor that combines an extracellular antigen-recognition domain, typically derived from a monoclonal antibody, with intracellular T-cell activation and costimulatory signaling domains. This design enables MHC-independent recognition of tumor-associated antigens and induces potent T-cell activation, proliferation, and targeted cytotoxicity against antigen-expressing malignant cells [43,44].

CAR T-cell therapy revolutionized the treatment paradigm of R/R B-ALL. This form of immunotherapy is based on autologous T lymphocytes that are genetically engineered and activated ex vivo to recognize and eliminate malignant blasts through specific targeting of surface antigens expressed on leukemic cells. For adults patients with B-ALL, the three main CAR-T products—tisagenlecleucel (tisa-cel), brexucabtagene autoleucel (brexu-cel), and obecabtagene autoleucel (obe-cel)—target CD19 but differ in key structural features: tisa-cel employs a 4-1BB costimulatory domain, brexu-cel uses a CD28 domain, and obe-cel incorporates a 4-1BB domain with optimized manufacturing processes designed to enhance T-cell expansion and persistence [45,46,47]. These structural distinctions influence kinetics of T-cell proliferation; persistence; and potentially toxicity profiles such as cytokine release syndrome (CRS) and neurotoxicity ICANS—immune effector cell-associated neurotoxicity syndrome. These two complications share a common pathogenic feature: endothelial injury. In CRS, the massive cytokine surge—particularly involving IL-6, IFN-γ, and TNF-α—triggers widespread endothelial activation, increased vascular permeability, and capillary leak, contributing to systemic inflammation and organ dysfunction. In ICANS, endothelial activation and blood–brain barrier disruption play a central role, with endothelial dysfunction leading to neuroinflammation, cerebral edema, and altered neurovascular homeostasis. Thus, endothelial injury represents a unifying mechanistic underpinning in the pathogenesis of both CRS and ICANS.

Below, we summarize the results of the main clinical studies, with particular emphasis on the role of CAR T-cell therapy as a bridge to allogeneic hematopoietic stem cell transplantation (Table 5). Tisa-cel was initially approved for pediatric and young adult R/R B-ALL based on the pivotal ELIANA trial, which demonstrated a CR rate of 82% and MRD negativity in most responders; 24-month OS reached 66%, with 62% of responders maintaining remission at 2 years. Real-world evidence from the Center for International Blood and Marrow Transplant Research (CIBMTR) registry (*n* = 255) confirmed these results, reporting a CR/CRi rate of 85.5%, 12-month duration of response (DOR) of 61%, and 12-month OS of 77.2%, highlighting reproducibility across clinical settings [48]. For adult patients, brexu-cel was evaluated in the Phase II ZUMA-3 trial, achieving a CR/CRi rate of 71% among 55 adults with R/R B-ALL and a median OS of ~26 months; updated analyses show a median OS of 47 months in responders. In a post hoc analysis, Shah et al. evaluated the impact of prior therapies and subsequent allo-HSCT. Overall, 14 of 57 patients (25%) who underwent prior therapies or CR with incomplete hematologic recovery (CRi) proceeded to allo-HSCT. Seven out of 14 patients transplanted (50%) remained in ongoing remission, one patient (7%) experienced relapse, and five patients (36%) had died. The median duration of remission (DOR) was longer in patients who received allo-HSCT compared with those who did not (44.2 vs. 18.6 months), whereas OS was comparable between groups (47.0 months with allo-HSCT vs. not reached in non-transplanted patients) Real-world registries confirm high CR/CRi (~80%), MRD negativity, 6-month RFS (~55%), and OS (~80%). In a subset (~15%) of patients, brexu-cel was used as a bridge to allo-SCT, with 1-year event-free survival (EFS) and OS of 63% and 74%, respectively, and NRM 20%, demonstrating feasibility of sequential CAR-T and transplant [49,50].

Obe-cel, a newer 4-1BB CAR-T with optimized manufacturing, was evaluated in the Phase Ib/II FELIX trial, showing an overall response rate of 76.6%, CR of 55.3%, median OS of 15.6 months, and high rates of MRD negativity. Notably, only a minority (~18%) required subsequent allo-SCT by ~21.5 months, suggesting durable remissions in some patients without immediate transplant [49]. Retrospective single-center analyses support CAR-T as a bridge to allo-HSCT: in 50 adult R/R B-ALL patients receiving CAR-T prior to allo-HSCT, 92% achieved CR/CRi, 1-year OS was ~80%, and 1-year EFS ~60%, with superior outcomes in patients achieving deep MRD negativity before transplant. Collectively, tisa-cel, brexu-cel, and obe-cel induce profound remissions with high MRD-negativity rates, supporting their use as standalone salvage therapy or as a bridge to allo-SCT in appropriately selected patients with R/R B-ALL [51,52,53].

## 6. Ph-like ALL

Ph-like ALL is a molecularly defined high-risk B-ALL subtype characterized by a gene expression profile similar to Philadelphia chromosome-positive ALL but lacking the BCR::ABL1 fusion gene [54,55,56]. Ph-like ALL harbors several genetic alterations that activate cytokine receptor and kinase signaling pathways, including CRLF2 rearrangements, JAK-STAT pathway mutations, ABL-class fusions (ABL1, ABL2, and PDGFRB), and frequent deletions of lymphoid transcription factors such as IKZF1; this genetic complexity defines intrinsic chemoresistance and aggressive clinical behavior.

Patients with Ph-like ALL can be identified using several complementary approaches [57]. Gene expression profiling (GEP) remains the gold standard for initial classification, though it is not routinely available in all clinical laboratories. Targeted RNA sequencing and fusion panels are increasingly employed in clinical practice to detect kinase fusions. Cytogenetics and FISH may reveal known rearrangements but have limited sensitivity for cryptic or novel fusions. Next-generation sequencing (NGS) panels can identify mutations in JAK-STAT or RAS pathway genes and provide a useful complement to RNA-based methods. Finally, single-nucleotide polymorphism (SNP) arrays detect genomic abnormalities affecting key signaling pathways, including both copy number alterations and copy-neutral loss of heterozygosity (CN-LOH), offering additional insights into the genomic landscape; however, this technique is not yet widely available in routine clinical settings. A qPCR-based assay, such as the *BCR-ABL1-like predictor* developed by Chiaretti et al., enables rapid identification of Ph-like ALL cases. This approach, based on a set of gene expression markers, is low-cost and readily implementable in routine diagnostic practice [58].

Ph-like ALL is particularly prevalent in adolescents and young adults, with peak frequencies of approximately 25–30% in patients aged 21–39 years, compared with lower rates in children and older adults. Ph-like ALL is characterized by adverse clinical features, including a higher likelihood of persistent MRD positivity after induction chemotherapy and inferior event-free and overall survival relative to non-Ph-like B-ALL [59,60].

In the trial GIMEMA LAL1913, the application of “BCR/ABL1-like predictor” allowed the researchers to identify Ph-like ALL cases in roughly one-third of adult B-ALL cases. This subgroup showed significantly lower CR rates (74.1% vs. 91.5%) and markedly higher rates of MRD positivity at post-induction decision points compared with non-Ph-like ALL (52.9% vs. 20%). Similarly, Ph-like cases demonstrated inferior EFS and DFS (33.5% vs. 66.2% at 24 months) [61]. Current standard therapy for Ph-like ALL remains intensive chemotherapy followed by allo-HSCT in CR1 mostly for patients with persistent MRD or other high-risk features, as long-term outcomes with chemotherapy alone are suboptimal. The presence of actionable kinase alterations has introduced targeted therapies—particularly tyrosine kinase inhibitors (TKIs) for ABL-class fusions or JAK-STAT pathway lesions—and opened the way to clinical trials in which several targeted drugs are assimilated into frontline and salvage strategies. However, the optimal integration of such agents and their impact on reducing transplant reliance remain areas of active investigation. Immunotherapy with blinatumomab has been evaluated within this high-risk context. A post hoc analysis of the Phase III TOWER trial comparing blinatumomab with standard-of-care chemotherapy in adults with R/R B-ALL identified a Ph-like signature in ~11% of sequenced patients; among these, blinatumomab significantly improved median OS compared with chemotherapy, and outcomes in blinatumomab-treated Ph-like patients were comparable to those of non-Ph-like patients, suggesting that blinatumomab may mitigate some of the adverse prognostic impact of the Ph-like genotype [62]. In the GIMEMA LAL2317 phase 2 study, which enrolled adult Ph-negative B-ALL, including 31 Ph-like cases, the addition of blinatumomab to intensive chemotherapy increased MRD negativity from 72% to 93% overall; however, among patients achieving MRD negativity at a defined time point, the cumulative incidence of relapse was higher in Ph-like patients (42.5% vs. 17.5%), indicating that blinatumomab plus chemotherapy alone may be insufficient to fully counteract the negative biological influence of Ph-like alterations and that additional targeted approaches (e.g., TKIs) may be required for optimal disease control [28]. The integration of CAR T-cell therapies in Ph-like ALL has also been described: retrospective analyses suggest that CAR-T followed by allo-HSCT yields comparable 3-year overall survival and relapse-free survival in Ph-like patients compared with Ph+ and other high-risk ALL subsets, with high rates of MRD-negative response after CAR-T infusion [63]. Despite these advances, there is no consensus yet that achieving MRD negativity with frontline targeted or immunotherapeutic regimens can safely obviate the need for allo-HSCT in Ph-like ALL. Current evidence continues to support allo-HSCT in CR1 for most patients with persistent MRD or adverse genetic features; however, ongoing studies are refining risk stratification and exploring whether subsets of patients with deep early MRD response might benefit from alternative consolidation strategies.

## 7. Ph-Positive ALL

Ph-positive ALL accounts for approximately 20–30% of adult B-cell ALL cases, with increasing incidence with age, and has historically been associated with a particularly unfavorable prognosis [64]. In the pre-TKI era, long-term survival rates were poor, and allo-HSCT in CR1 represented the only curative strategy for eligible patients. Early study groups consistently demonstrated superior outcomes for transplanted patients compared with those treated with chemotherapy alone, establishing allo-HSCT as standard consolidation for virtually all fit Ph+ ALL patients [65,66,67,68]. In Ph-positive ALL, consistently with the introduction of the target therapy (imatinib in 2000 and dasatinib in 2006 and ponatinib in 2010), two major strategies have been used which revolutionize allo-HSCT indication. The first consists of a chemo-free approach, and the second involves the combination of TKIs and immunotherapy with blinatumomab. These two strategies have been strengthened by the ability to monitor treatment response through MRD assessment, thereby enabling more informed and response-adapted therapeutic decision-making. The GIMEMA group was the pioneer of the chemo-free approach. Since 2004, all GIMEMA trials have been designed with an induction-phase chemo-free with corticosteroids associated with central nervous system prophylaxis. This approach dramatically reduced early mortality and achieved a CR rate around 100% [69,70,71]. In parallel, the experiences from MDACC demonstrated the efficacy of chemotherapy backbones (low or high intensity) combined with second- and third-generation TKIs. A comparative analysis showed superior molecular response rates and RFS with ponatinib versus dasatinib when combined with low-intensity chemotherapy, challenging the paradigm of universal transplant in CR1 [72]. The deepest response in terms of MRD negativity was observed with ponatinib, a pan-BCR::ABL1 inhibitor, able to overcome T315I mutation resistance. Long-term follow-up of a phase II study combining hyper-CVAD with ponatinib reported 5-year OS rates approaching 75%, with durable molecular remissions and a significantly reduced need for allo-HSCT in patients achieving deep MRD negativity [73]. Building on these findings, the Spanish PONAFIL trial evaluated frontline ponatinib combined with standard induction and consolidation chemotherapy followed by allo-HSCT. In this study, allo-HSCT was performed in 26 of 30 patients, with 3-year EFS of ~70% and OS of ~96% (median follow-up 2.1 years), demonstrating both the feasibility and the survival benefit of integrating ponatinib as a bridge to transplant consolidation [74]. Indeed, the PhALLCON Phase III trial compared frontline ponatinib versus imatinib, each combined with reduced-intensity chemotherapy. Ponatinib achieved higher rates of MRD-negative complete remission at the end of induction (≈34% vs. 17% with imatinib) and more durable remissions, resulting in a lower proportion of patients proceeding to allo-HSCT relative to imatinib [75]. Regarding the combination of TKIs plus blinatumomab, the GIMEMA LAL2116 D-ALBA protocol was designed with induction chemo-free in which dasatinib was administered plus glucocorticoids, followed by at least two cycles of blinatumomab as consolidation. Sixty-three patients were enrolled, and the CR rate resulted in 98%. Notably, molecular responses increased from approximately 29% following dasatinib alone at the end of induction to 60% after two cycles of blinatumomab, translating into excellent clinical outcomes with a median follow-up of 18 months: OS and DFS rates of 95% and 88%, respectively [76]. A sub-analysis of D-ALBA identified patients with IKZF1^plus^ aberrations (deletion of IKZF1 plus deletion of CDKN2A-2B and/or deletion of PAX5) as having inferior outcomes, consistent with the negative prognostic impact of such high-risk genomic features. Short et al. evaluated a sequential regimen of blinatumomab with ponatinib in a Phase II trial at MDACC, reporting complete molecular remission (CMR) rates of 64% after one course and 85% upon treatment completion; using highly sensitive NGS (10^−6^), MRD negativity was confirmed in approximately 88% of cases [77]. Building on these immunotherapy-enhanced strategies, the GIMEMA study group has advanced to a Phase III comparison (GIMEMA LAL2820) of frontline ponatinib plus blinatumomab versus imatinib plus chemotherapy in newly diagnosed adult Ph-positive ALL. The first results from this trial were presented at the 67th American Society of Hematology (ASH) Annual Meeting, showing that the chemo-free regimen significantly improves efficacy endpoints, with a CR rate of ~94% in the experimental arm (imatinib plus chemotherapy) and favorable survival outcomes when compared with standard TKI plus chemotherapy [78]. These data support a shift toward ponatinib plus immunotherapy as a potential new standard of care in Ph-positive ALL and further complicate the previously more straightforward indication for allo-HSCT, which is increasingly being tailored based on early MRD dynamics and adverse biological features. A further critical advance has been in prognostic stratification. The identification of adverse genomic signatures, such as IKZF1^plus^, combined with the widespread adoption of high-sensitivity NGS MRD assays, allows for more precise discrimination of patients at risk of relapse. These tools guide decisions regarding allo-HSCT: patients with sustained deep MRD negativity may safely defer transplant, whereas those with adverse genomic features or persistent sub-clinical disease remain candidates for transplant consolidation. Collectively, these advances—progressing from TKI-based therapy to TKI plus immunotherapy to refined molecular stratification and deep MRD monitoring—have transformed the management of Ph-positive ALL. The role of allo-HSCT has evolved from a universal consolidation strategy to a selectively applied, risk-adapted intervention tailored to disease biology and depth of response.

## 8. Discussion

In an era in which the transplant procedure improved with a reduction of TRM, the role of allo-HSCT itself has been profoundly reevaluated across multiple hematologic malignancies, including B-ALL. Consistently with a predominant role of MRD with deeper technique, the introduction of immunotherapy contributed to improve the outcome and reshape the indication to allo-HSCT [77]. Indeed, the prognostic stratification contributes to refine a better patient selection. The MRD-driven consolidation therapy emerged as the most important tool for Ph-negative B-ALL. Current recommendations in frontline therapy propose allo-HSCT primarily for patients who remain MRD-positive at the end of induction, irrespective of baseline biological risk, whereas MRD-negative patients can achieve durable remission with immunotherapy consolidation using blinatumomab. In the R/R setting, a growing possibility of immunotherapies—including bispecific T-cell engagers, antibody–drug conjugates, and CAR T-cell therapy—has demonstrated the capacity to induce complete and molecular remissions, serving as effective bridges to transplant [78]. Notably, preliminary evidence suggests that CAR T-cell therapy may in some cases induce durable remissions without the need of allo-HSCT; however, these findings remain preliminary and not yet supported by robust evidence or consensus. The Ph-like subgroup remains particularly challenging. Despite significant advances in the molecular understanding and diagnostic identification of distinct driver lesions, these patients are often unable to achieve MRD negativity with standard chemotherapy or immunotherapy, remaining persistently MRD-positive [79]. Enhanced knowledge of the underlying molecular pathways has prompted extensive clinical investigation into targeted therapies, typically in combination with frontline chemotherapy. Ongoing trials will clarify whether subsets of Ph-like patients can be consolidated with immunotherapy and potentially avoid allo-HSCT. Finally, the Ph-positive B-ALL is the subgroup who benefitted from recent strategy and therapeutic innovations. Since the introduction of imatinib, treatment paradigms have shifted from considering allo-HSCT as universal consolidation for all patients toward a more individualized selection of this therapeutic strategy [79]. Efforts to optimize chemo-free regimens with TKI plus blinatumomab have led to high rates of deep molecular remission and significant reductions in the need for routine transplant. Additional advances in prognostic stratification (e.g., IKZF1^plus^) and the use of NGS for MRD monitoring now enable tailored decisions regarding the necessity of allo-HSCT, reserving transplant for patients with adverse genomic signatures or persistent MRD [80,81,82]. Overall, across B-ALL subtypes, the past 20 years have witnessed a paradigm shift in transplant strategy, from universal application to risk-adapted, MRD-driven approaches, combined with the integration of targeted and immunotherapeutic agents. These advances have not only improved long-term outcomes but also allowed for a more personalized selection of patients for whom allo-HSCT remains truly beneficial.

## 9. Conclusions

In this review, we aimed to delineate how the role of allo-HSCT has evolved across the distinct biological subtypes of adult B-ALL in the era of precision medicine and immunotherapy. The integration of refined risk stratification, MRD-driven decision-making, and highly effective immunotherapeutic approaches has translated into improved transplant selection and overall outcomes. Nevertheless, disease relapse following allo-HSCT and CAR T-cell therapy remains the leading cause of treatment failure. Increasing evidence supports the central prognostic value of peri-transplant MRD assessment, both before and after cellular therapies, to identify patients at highest relapse risk. Consequently, the development of post-transplant and post-CAR T-cell preventive strategies—including targeted maintenance, immune modulation, and MRD-guided interventions—represents a critical unmet need [83]. Future efforts should focus on optimizing individualized maintenance approaches capable of preserving GvL activity while minimizing toxicity and immune-related complications.

## Figures and Tables

**Table 3:** Main clinical trials of blinatumomab in B-ALL.

Authors	Study Type	*n* Patients	Disease Status at Blinatumomab	OS	DFS/RFS	GRFS	% Proceeding to Allo-HSCT	TRM/NRM
Kantarjian et al. [26]	Phase III RCT	405	R/R B-ALL	OS superior vs. chemo	NA	NA	NA	NA
Dombret et al. [29]	Phase II	113	MRD + CR1/CR2	Median OS 36.5 mo	Median RFS 18.9 mo (23.6 mo in MRD responders)	NA	67%	NA
Gökbuget et al. [30]	Phase III RCT	405	R/R Ph − B-ALL	Median OS 7.7 mo	NA	NA	~34% (65 pts)	NA
Fu et al. [31]	Single-center case series	3	MRD + adult B-ALL	NR	2 yr DFS ≈67%	NA	100%	0 severe AEs
Sayyed et al. [32]	Retrospective cohort	NR	Adult B-ALL pre-HSCT	2 yr OS 65.4%	NA	42.2%	100%	NRM 3.2%
Llaurador et al. [33]	Retrospective pediatric cohort	31	Pediatric/AYAs B-ALL	NR	Improved LFS vs. controls	NA	100%	NA
Algeri et al. [34]	Multicenter pediatric cohort	78	Pediatric/AYAs B-ALL pre-HSCT	2 yr OS 89.2%	2 yr DFS 72.2%	NA	~100%	NRM 2.6%

ALL, acute lymphoblastic leukemia; AYAs, adolescents and young adults; CR, complete remission; DFS, disease-free survival; GRFS: graft-versus-host disease-free, relapse-free survival; HSCT, hematopoietic stem cell transplantation; LFS, leukemia-free survival; MRD, minimal measurable disease; NA, not available; NRM, no-relapse mortality; OS, overall survival; Ph, Philadelphia; R/R, relapsed/refractory; RCT, randomized controlled trial; RFS, relapse-free survival.

**Table 4:** Main clinical trials of inotuzumab ozogamicin as a bridge to Allo-HSCT.

Authors	Study Type	*n* Patients	Disease Status at InO	OS	DFS/RFS	GRFS	% Proceeding to Allo-HSCT	TRM/NRM
Kantarjian et al., 2016 [36]	Phase III RCT	326 (InO arm *n* ≈ 164)	R/R B-ALL	Median OS 7.7 mo (InO); 1-yr OS NR	NA	NA	~41%	SOS/VOD post-HSCT ~14–18%; TRM NR
Kantarjian et al., 2021 [37]	Post-hoc/transplant-focused analysis	NR	R/R B-ALL	NR	NA	NA	NA	Increased SOS/VOD esp. >2 InO cycles; NRM NR
Kayser et al., 2023 [39]	Retrospective registry	58	R/R B-ALL, CR pre-HSCT in 84%	Median OS 11.2 mo; 1-yr 50%; 2-yr 36.7%	NA	NA	100% (selected transplanted cohort)	SOS/VOD 29%; high fatality among cases
Kondo et al., 2025 [40]	Retrospective	NA	R/R ALL/MPAL	NA	NA	NA	NA	NA
Gökbuget et al., 2020 [29]	Retrospective	NA	R/R B-ALL	2-yr OS 63.0% (vs. 31.2% standard)	2-yr DFS 49.6% (vs. 22.9%)	NA	77.3% CR pre-HSCT	Trend toward lower NRM
Stelmach et al., 2020 [42]	Retrospective single-center	NA	R/R B-ALL	NA	NA	NA	NA	NA

ALL, acute lymphoblastic leukemia; CR, complete remission; DFS, disease-free survival; GRFS, graft-versus-host disease-free, relapse-free survival; HSCT, hematopoietic stem cell transplantation; NA, not available; NRM, no relapse mortality; OS, overall survival; R/R, relapsed/refractory; RCT, randomized controlled trial; RFS, relapse-free survival.

**Table 5:** Main clinical trials of CAR-T in B-ALL.

Authors	Study Type	*n* Patients	Disease Status at CAR-T	OS	DFS/RFS	% Proceeding to Allo-HSCT
Davila et al., 2014 [43]	Real-world registry	255	R/R B-ALL	12 mo OS 77.2%	12 mo DOR 61%	NR
Maude et al., 2018 [45]	Phase II	55	Adult R/R B-ALL	Median OS ~26 mo; responders 47 mo	Median DOR NR	~25%
Roddie et al., 2024 [47]	Phase Ib/II	127	Adult R/R B-ALL	Median OS 15.6 mo	NR	~18%
Shah et al., 2025 [50]	Post hoc analysis	57	CR/CRi post brexu-cel	OS 47.0 mo (allo-HSCT) vs. NR (no HSCT)	DOR 44.2 mo (allo-HSCT) vs. 18.6 mo	25% (14/57)
Retrospective series [49,50]	Retrospective	50	Adult R/R B-ALL	1 yr OS ~80%	1 yr EFS ~60%	100% (selected cohort)

ALL, acute lymphoblastic leukemia; CR, complete remission; DFS, disease-free survival; DOR, duration of remission; HSCT, hematopoietic stem cell transplantation; NR, not reached; OS, overall survival; R/R, relapsed/refractory; RFS, relapse-free survival.

## Data Availability

No new data were created or analyzed in this study. Data sharing is not applicable to this article.
